# Biofabrication of ZnO/Malachite nanocomposite and its coating with chitosan to heal infectious wounds

**DOI:** 10.1038/s41598-022-15768-5

**Published:** 2022-07-08

**Authors:** Zahra Rajabloo, Mohammad Reza Farahpour, Parvaneh Saffarian, Saeed Jafarirad

**Affiliations:** 1 Department of Biology, Science and Research Branch, Islamic Azad University, Tehran, Iran; 2 Department of Clinical Sciences, Faculty of Veterinary Medicine, Urmia Branch, Islamic Azad University, Urmia, Iran; 3 Department of Organic and Biochemistry, Faculty of Chemistry, University of Tabriz, Tabriz, Iran; 4 Research Institute of Bioscience and Biotechnology, University of Tabriz, Tabriz, Iran

**Keywords:** Biochemistry, Microbiology, Health care, Medical research, Infectious diseases, Skin diseases, Trauma, Environmental chemistry

## Abstract

Recently, nanocomposites produced from clays and metals coated with chitosan have shown wound healing activity. This study aimed to synthesize the zinc oxide/malachite nanocomposite (ZnO/Mlt-NC) and its coating form with chitosan (ZnO/Mlt/Chsn-NC). Physicochemical characterization of the produced nanocomposites was investigated. Biomedical effects of nanocomposites, such as in vivo and in vitro antibacterial activity, antioxidant properties, cytotoxicity, and modulation in the gene expressions of interleukin-1β (IL-1β), interleukin-6 (IL-6), interleukin-10 (IL-10), and transforming growth factor-β (TGF-β) and histopathological parameters, were also investigated. Expression intensities of basic fibroblast growth factor (bFGF) and tumor necrosis factor alpha (TNF-α) were also investigated by immunofluorescence staining. To investigate biomedical effects under in vivo conditions, infected wounds were induced and inoculated with *Staphylococcus aureus* (ATCC 25923), and *Pseudomonas aeruginosa* (ATCC 27853). The results indicated spherical ZnO nanoparticles on the surface of malachite and strong antibacterial activity and antioxidant properties. The ointments produced from the nanocomposites also exhibited wound healing activity. The administration of the ointments prepared from ZnO/Mlt, and ZnO/Mlt/Chsn NCs decreased the expressions of IL-1β, IL-6, and TNF-α, while it increased the expressions of IL-10, TGF-β and bFGF. In sum, the nanocomposites produced from ZnO, malachite, and chitosan had better biological activity than ZnO/Malachite nanocomposites. We suggest applying ZnO/Mlt/Chsn nanocomposites in the structure of ointments to treat infected wounds after future clinical studies.

## Introduction

Skin wounds cause damage to healthcare systems and loss economic^1^. Wounds are classified as acute and chronic based on the pathogenesis and consequences^2^. Acute wounds induce molecular processes to obtain structural integrity. Immune cells and factors play pivotal roles in acute wound healing^3^. The faulted regulation of the immune response results in the formation of chronic wounds^4^. Infectious wounds are a form of acute wounds characterized by the presence of bacteria in viable tissue and damage to tissues^5^. The infections start with bacteria colonization and can cause systemic infection. *Staphylococcus aureus* and *Pseudomonas aeruginosa* are the most common bacteria in infected wounds^6^. In infected wounds, the wound healing process is delayed^7^. Infected wounds also cause overproduction of reactive oxygen species and induce faults in antioxidant systems^8^. Antibiotics are used to treat infected wounds; however, they cause antimicrobial resistance. Therefore, it is essential to find safe and novel agents for the treatment of infected wounds^9^.

Metal oxides nanomaterials are safe and cheap structures used to treat wounds^10^. Copper oxide is extensively used for wound healing activity owing to its biomedical properties like antibacterial activity^3^. Malachite is a form of copper oxide ore derivatives as Cu_2_CO(OH)_2_^11^. It has been reported that malachite exhibits antibacterial activity against *S. aureus* and *P. aeruginosa* by changing the integrity of the cell membrane and antioxidant properties^12^. Recently, studies have reported synergistic interaction effects between clays and metal nanoparticles^13–15^. Metallic nanoparticles, including ZnO nanoparticles (ZnONPs), are extensively used in medicine. ZnONPs exhibit antioxidant properties via the electron donation property of the oxygen atom in ZnO nanomaterial and antibacterial properties via the electrostatic activity between the negative charge of bacterial cells and the positive charge of ZnO nanoparticles that can be used in the wound healing process^16^. ZnONPs also influence oxidative stress and increase the generation of reactive oxygen species^16^. They also promote the regeneration of damaged tissues by activating collagen synthesis^17^. Metal oxide NPs, including ZnO NPs, may be stabilized by being mixed with other inorganic structures like malachite^18,19^. This study aims to synthesize ZnO/Mlt-NC as an agent for the wound healing process using the external biofabrication method. This method requires plant active compounds, including phenolic compounds for capping and stabilizing^13–15^. In this study, we used pennyroyal (*Mentha pulegium)* extract to synthesize ZnO/Mlt-NC as a capping and stabilizing agent. *M. pulegium* belongs to the mint family, Lamiaceae. It contains some phenolic compounds, such as syringic acid, ferulic acid, and flavonoid compounds, including isorhamnetin-3-O-glucoside and kaempferol-3-O-rutinoside that can contribute to capping and stabilizing. Therefore, in the present study, for the first time, *M. pulegium* extract was used to synthesize ZnO/Mlt-NC^20^.

To improve biological properties, the synthesized nanocomposites were coated with chitosan. Chitosan is known to have antibacterial^21^ and anti-inflammatory^22^ properties and exhibits skin regenerative behavior, biocompatibility, and biodegradability^14,15,23^. It is also a safe compound and has very low toxicity^21^. It shows antibacterial activity via penetration in the cellular membrane^21^ and clears free radicals via adsorption, ion-exchange, and chelation^13^.

In this study, ZnO/malachite nanocomposites coated with chitosan (ZnO/Mlt/Chsn-NC) were synthesized to heal infected wounds in a mouse model. In addition, not only the structural properties of ZnO/Mlt/Chsn-NC were investigated, but also the efficiency of ZnO/Mlt/Chsn-NC and ZnO/Mlt-NC was evaluated. Furthermore, antibacterial activity, antioxidant properties, and the genes expression of interleukin-1β (IL-1β), interleukin-6 (IL-6), interleukin-10 (IL-10), transforming growth factor-β (TGF-β), basic fibroblast growth factor (bFGF), and tumor necrosis factor alpha (TNF-α) were investigated.

## Results and discussion

**Figure 1:**
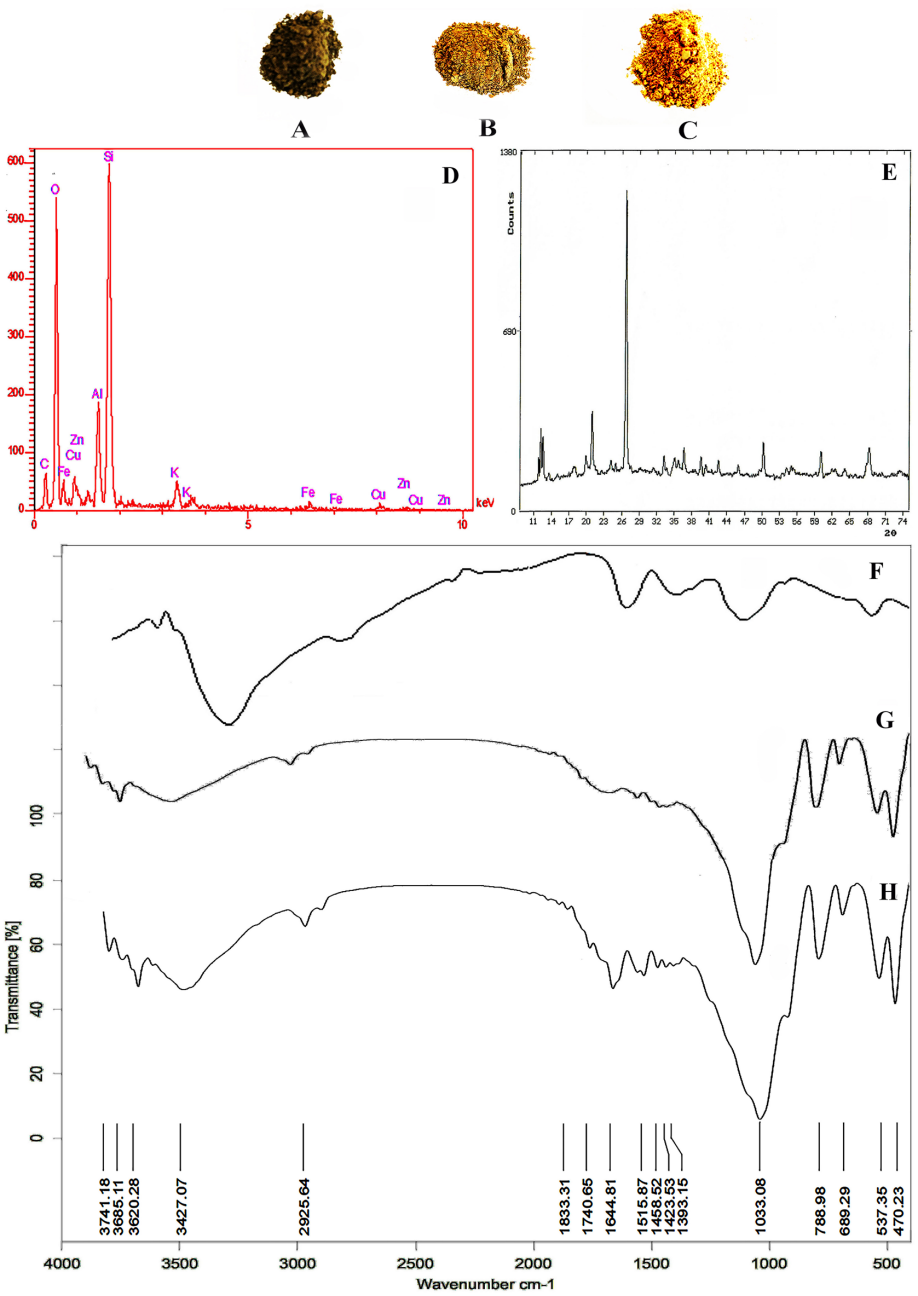
The physical form of (**A**) Mlt; (**B**) ZnO/Mlt-NC; (**C**) ZnO/Mlt/Chsn-NC; (**D**) EDX pattern of ZnO/Mlt-NC; (**E**) XRD diffractogram of ZnO/Mlt-NC; FTIR patterns of (**F**) Chsn; (**G**) ZnO/Mlt-NC; (**H**) ZnO/Mlt/Chsn-NC.

Figure 1A–C shows the physical form of Mlt, ZnO/Mlt-NC and ZnO/Mlt/Chsn-NC, respectively.

### Structural characterization of the prepared nanocomposites

Malachite contains SiO_2,_ Cu(CO_3_)_2_, Cu(OH)_2_, Fe_2_O_3_, Al_2_O_3_, and other metallic oxides, such as Na, Ca, K and Mg oxides^11^. Figure 1D shows the presence of Cu, Zn, Si, Fe, Al, K, C and O elements in the as-fabricated ZnO/Mlt-NC. Some parts of the C element originate from secondary metabolites of the extract connected to NC^13–15^.

The results of the XRD pattern in Fig. 1E exhibited multiple obvious peaks for the malachite-based composite, which are similar to the peaks reported in previous studies^11^. In addition to malachite, a small amount of quartz was also observed. The results indicated strong and sharp diffraction lines originated from ZnO with a hexagonal structure. The peaks observed at 31.76°, 34.42°, 36.25°, 47.53°, 56.59°, 62.85°, 67.94° and 69.08° show (100), (002), (101), (102), (110), (103), (112) and (202) planes, respectively. The results demonstrate the presence of ZnO nanoparticles, being in accordance with the standard spectrum of the JCPDS file No. 01-079-0206 (Fig. 1E)^24^. The crystalline structure of malachite was detected by the diffraction at 12.86°, 15.42°, 18.01°, 24.91°, 29.19°, 30.11°, 32.25°, 33.42°, 38.21°, 42.19°, 43.78°, 47.25°, 54.90° and 63.58°. The crystalline structure of quartz was detected by the sharp diffraction at 20.6°, 26.3°, 39.8°, 50.2°, and 60.1° according to the JCPDS card No. 46–1045 (Fig. 1E). The average crystallite size of ZnO nanoparticles was measured by the Debye–Scherrer formula as 19.13 nm.

**Figure 2:**
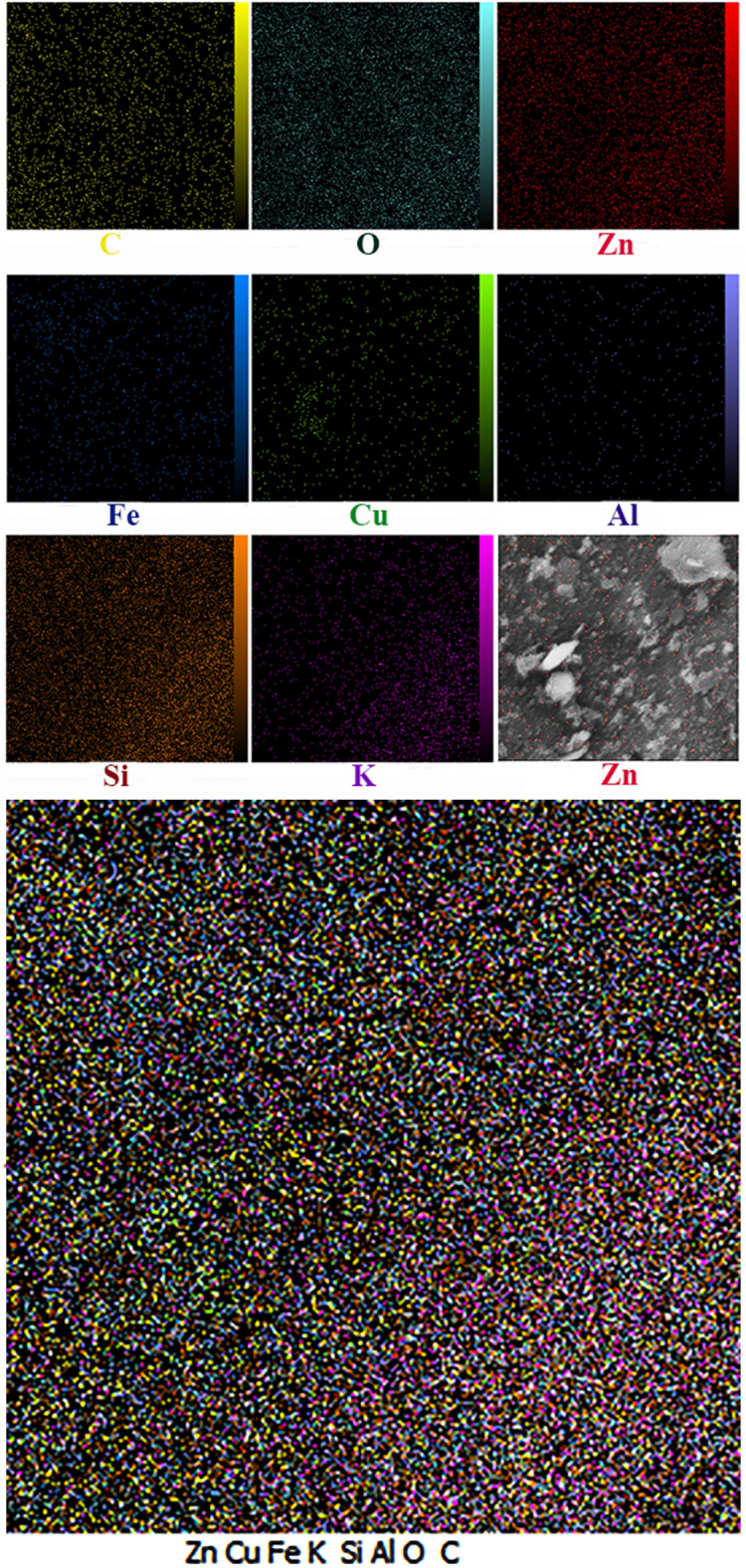
The FESEM/Map images of ZnO/Mlt-NC.

Figure 2 depicts the FESEM/Map of as-synthesized nanostructures. Based on the findings, ZnO nanoparticles completely coat malachite plates. The red spherical globules immobilized on malachite were observed in FESEM/Map images.

### FTIR characterization

The ZnO/Mlt-NC produced in this research showed stability due to in situ bio-capping by the phytochemical metabolites in pennyroyal derivations. The results of FTIR confirm the results (Fig. 1F–H). The stability is due to the presence of metabolites in pennyroyal derivations, such as syringic acid, ferulic acid, isorhamnetin-3-O-glucoside, and kaempferol-3-O-rutinoside^20^. The results of the FT-IR spectrum of ZnO/Mlt-NC showed three classes of bands. The first bands were peaks on 470.90, 535.96, 690.34 and 785.87 cm^−1^, which are related to Si–O, Cu–O, Al–O, and Fe–O stretching bands, respectively. The second bands were observed at 1034.56, 1423.19, 1642.97 and 1726.73 cm^−1^ that are assigned to C–O, C–C, C=C, and C=O of the aromatic rings of phenolic compounds and the carbonyl functional group, respectively. The band might be associated with functional groups of secondary metabolites in the plant extract attached to NCs^25^. The secondary metabolites of the extract, such as syringic acid and ferulic acid containing C–O, C=C and C=O groups, act as capping and stabilizing agents. It could be attributed to the secondary metabolites of the extract with significant affinity for attaching to metals. This prevents the agglomeration of ZnO/Mlt-NC. The third peak is observed at 3417.38 cm^−1^ that is due to isorhamnetin-3-O-glucoside and kaempferol-3-O-rutinoside. The peaks observed at 3620.74, 3691.53, 3739.34 and 3838.16 cm^−1^ are assigned to Si–O–H, Cu–OH, Al–O and Fe–O–H groups of ZnO/Mlt-NC (Fig. 1G)^24^. Figure 1F shows the peak at 1644 cm^−1^ that is attributed to residual N-acetyl groups (C=O stretching of amide I)^13^. A band at 1558 cm^−1^ is observed for the N–H bending of the primary amine. The CH_2_ bending and CH_3_ symmetrical deformations were approved by bands found at around 1423 and 1393 cm^−1^, respectively. Figure 1H presents a mixture of characteristic peaks due to presence of ZnO/Mlt-NC and Chsn.

### Morphological images

**Figure 3:**
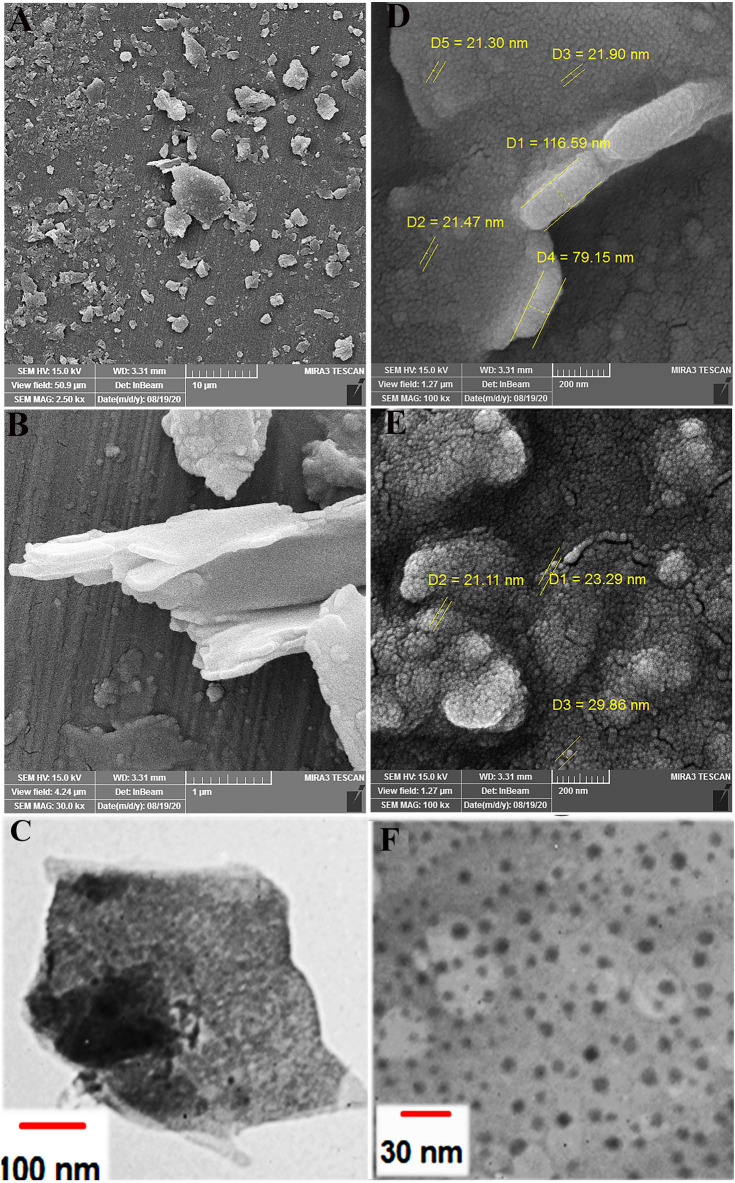
(**A**,**B**) FESEM images of malachite and (**D**,**E**) FESEM images of ZnO/Mlt-NC in different magnifications; (**C**) TEM image of malachite and (**F**) TEM image of ZnO/Mlt-NC.

Figure 3A,B and D,E depict the FESEM images of malachite and as-synthesized ZnO/Mlt-NC, respectively. According to Fig. 3D, the thickness of the malachite plane is 79–116 nm. Furthermore, it can be observed that ZnO nanoparticles have a globular shape. Based on the FESEM image, the size of as-synthesized ZnO particles on the malachite plate ranges from 21 to 29 nm. According to the TEM images in Fig. 3C,F, the black spherical globules of ZnO are immobilized on the malachite.

### Investigation on colloidal properties of ZnO/Mlt-NC

**Figure 4:**
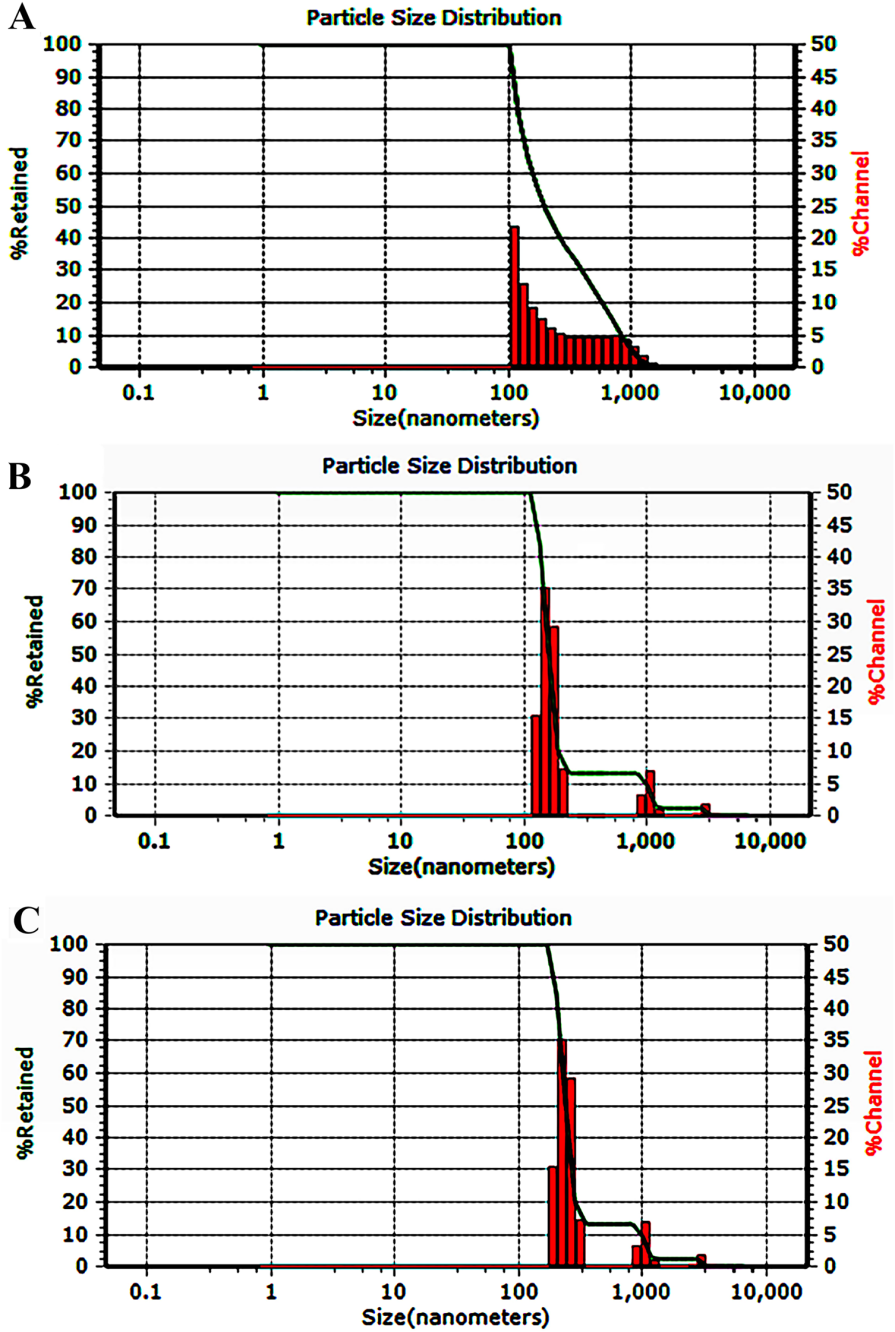
DLS patterns of (**A**) Mlt; (**B**) ZnO/Mlt-NC; and (**C**) ZnO/Mlt/Chsn-NC.

Figure 4 indicates that the D_max_ of the malachite, ZnO/Mlt-NC, and ZnO/Mlt/Chsn-NC was in the range of 102.2, 124.8, and 236.3 nm, respectively. The values obtained for the zeta potential of colloidal suspensions in the malachite, ZnO/Mlt-NC and ZnO/Mlt/Chsn-NC were found at + 17.2, + 18.9, and + 24.5 mV, respectively.

**Figure 5:**
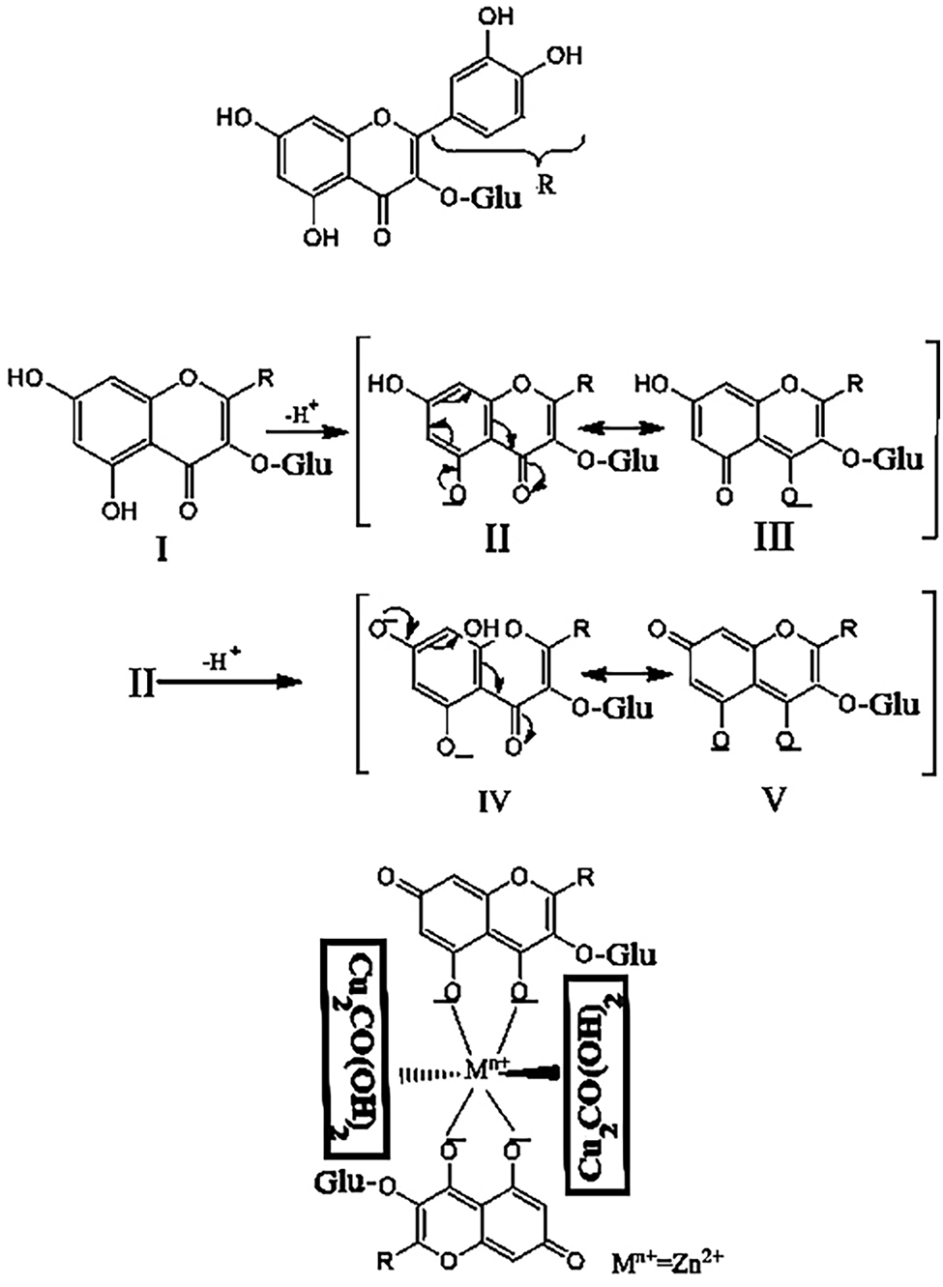
A proposed schematic mechanism of ZnO/Mlt-NC biofabrication based on isorhamnetin-3-O-glucoside in the plant extract.

Figure 5 proposed schematic mechanism of ZnO/Mlt-NC biofabrication based on isorhamnetin-3-O-glucoside in the plant extract.

### Antibacterial results

Table 1 presents the results of MIC and MBC. The lowest bacteriostatic and bactericide activities are observed in the Mlt group for both bacteria. ZnO/Mlt-NC exhibited better bacteriostatic and bactericide activities than the Mlt group did for both bacteria. The highest bacteriostatic and bactericide activities were observed for ZnO/Mlt/Chsn-NC for both bacteria. All the nanocomposites were efficient on *P. aeruginosa and S. aureus*. ZnO/Mlt/Chsn-NC showed better antibacterial activity than the antibiotics did in MIC and MBC tests.

**Table 1:** Bacteriostatic and bactericide activities of the prepared nanocomposites (µg/mL).

	*S. aureus*		*P. aeruginosa*	
	MIC	MBC	MIC	MBC
Mlt	52.50 ± 2.25	105.00 ± 7.65	105.00 ± 4.56	210.00 ± 4.62
ZnO/Mlt	2.25 ± 0.25	4.50 ± 0.12	3.5 ± 0.36	7.00 ± 0.25
ZnO/Mlt/Chsn	2.00 ± 0.13	4.00 ± 0.42	2.25 ± 0.15	4.50 ± 0.18
Bacitracin	4.20 ± 0.45	8.20 ± 0.85	–	–
Polymixin B	–	–	3.23 ± 0.4236	6.10 ± 0.25

**Figure 6:**
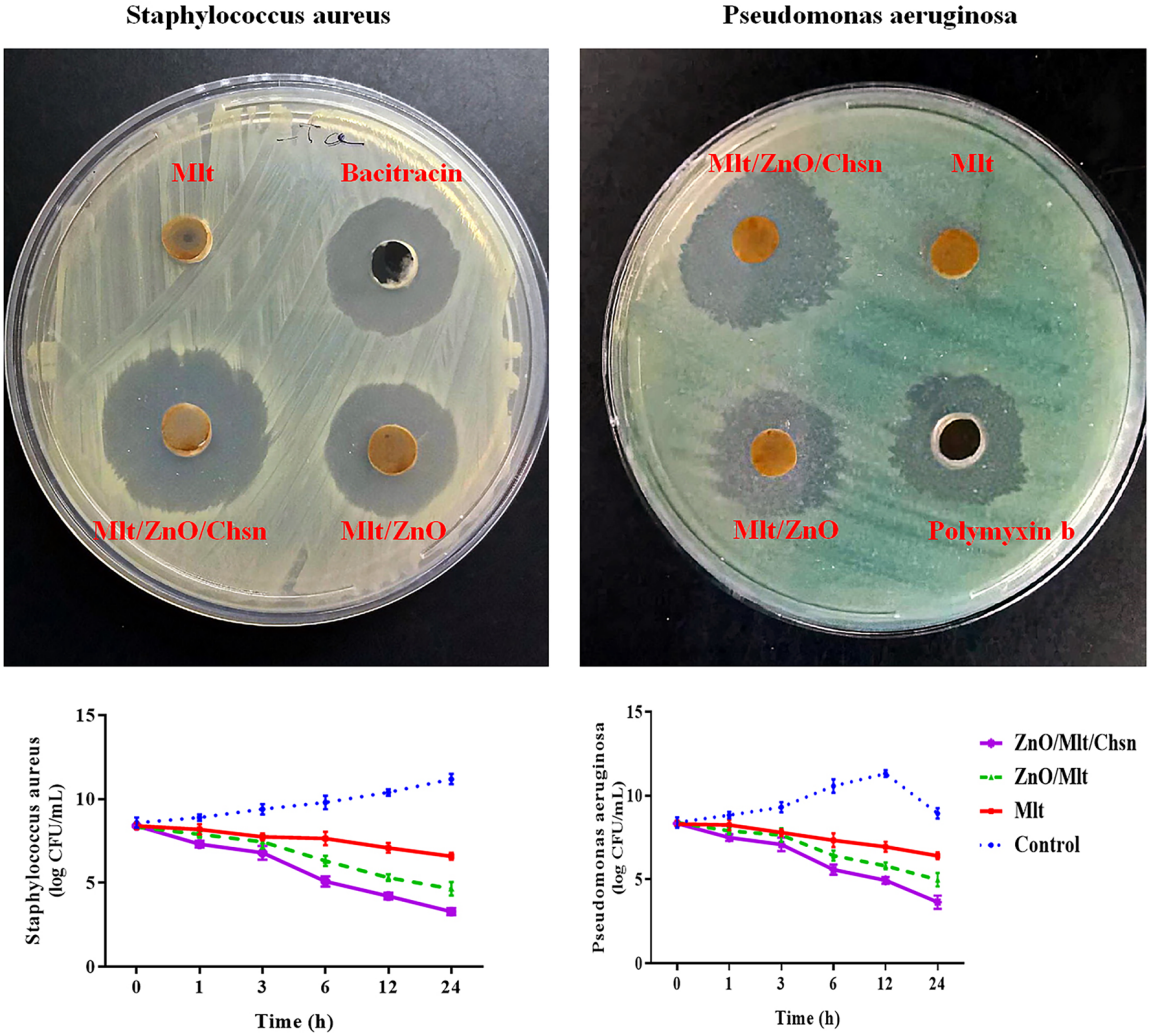
Antibacterial activity of Mlt, ZnO/Mlt-NC and ZnO/Mlt/Chsn-NC nanocomposites against *S. aureus* and *P. aeruginosa* in the well test. The charts show the kinetic of the antibacterial activity of experimental nanocomposites on *P. aeruginosa* and *S. aureus* and.

The results of the well test in Fig. 6 indicated that Mlt did not cause any inhibition zone for *P. aeruginosa*, but it formed a small inhibition zone for *S. aureus* (0.50 ± 0.707 mm). ZnO/Mlt nanocomposite had a higher inhibition zone than Mlt. The results revealed that ZnO/Mlt created an inhibition zone of 21.50 ± 1.41 mm for *S. aureus,* while it formed an inhibition zone of 18.50 ± 1.41 mm for *P. aeruginosa* (*P* = 0.021). The highest inhibition zone was observed in ZnO/Mlt/Chsn-NC than in the other nanocomposites. The results demonstrated that ZnO/Mlt/Chsn-NC created inhibition zones of 25.25 ± 2.12 mm and 23.25 ± 1.41 mm for *S. aureus*, and *P. aeruginosa* bacteria, respectively, which were significantly different(*P* = 0.0285). The diameters of the bacitracin antibiotic zone as a standard for *S. aureus* and the polymixin B antibiotic as a standard for *P. aeruginosa* were 20.10 ± 1.83 mm and 18.21 ± 1.12 mm for *S. aureus* and *P. aeruginosa* bacteria, respectively, which were significantly different (*P* = 0.0375). The results did not exhibit significant differences (*P* > *0.05*) between ZnO/Mlt/Chsn-NC and antibiotics for the zone diameter.

The results of the kinetic section demonstrated that all nanocomposites showed antibacterial activity from 1 to 24 h. Mlt, ZnO/Mlt, and ZnO/Mlt/Chsn nanocomposites demonstrated antibacterial activity at all times. The results of the kinetic time confirmed other antibacterial results. Based on kinetic findings, ZnO/Mlt/Chsn-NC exhibited the most powerful antibacterial activity, so that ZnO/Mlt/Chsn-NC decreased bacteria colony from 3 h after incubation, and the colony number was almost zero in 12 h after incubation; antibiotics showed similar effects after 24 h, and ZnO/Mlt and antibiotics exhibited similar effects.

**Figure 7:**
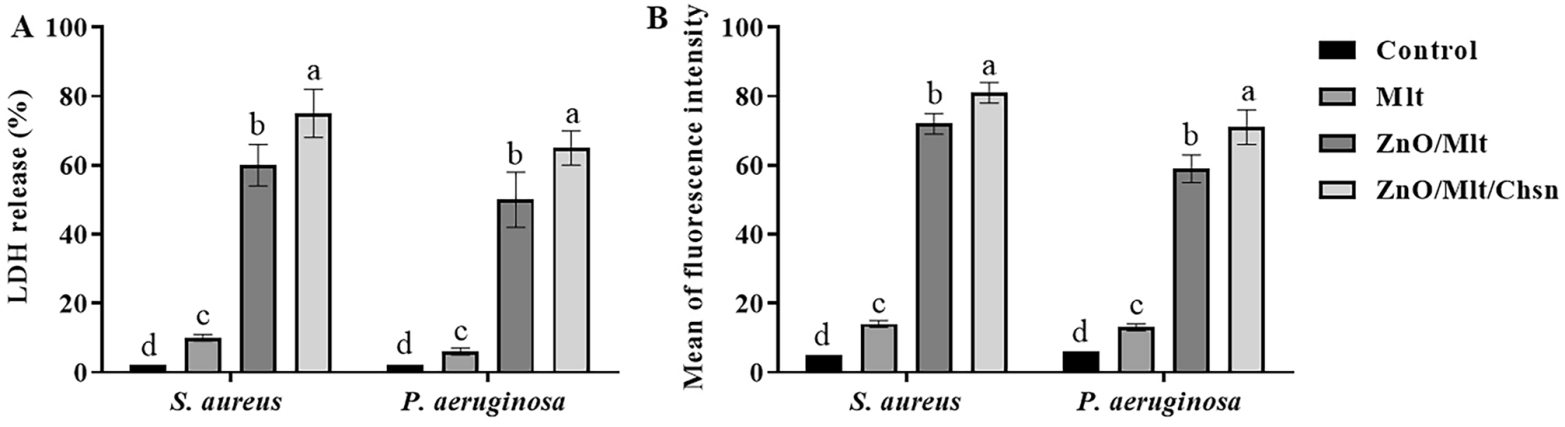
(**A**) Release of LDH (%) and (**B**) production of ROS of ZnO/Mlt/Chsn-NC nanocomposites on *Staphylococcus aureus* (*S. aureus*) and *Pseudomonas aeruginosa (P. aeruginosa)*.

As the results show, NCs exhibited antibacterial activity via the release of LDH and production of ROS (Fig. 7). The results showed that exposing bacteria to NCs increased the release of LDH compared to the control treatment (*P* = 0.0001). Production of ROS was greater in NCs than in the control treatment (*P* = 0.0001). Parallel with other findings, the greatest release of LDH was observed in bacteria exposed to ZnO/Mlt/Chsn, ZnO/Mlt, Mlt and control groups, respectively. Production of ROS was also greater in ZnO/Mlt/Chsn, ZnO/Mlt, Mlt and control groups.

The findings indicated that Mlt showed weak antibacterial properties. The results are similar to the ones reported by Anju et al.^12^. Malachite shows its antibacterial activity via the change in the integrity of the cell membrane and antioxidant properties^12^. The findings also revealed the antioxidant activity of Mlt in the DPPH test. Apparently, Mlt exhibited antibacterial activity in a longer time. The findings for the kinetic time indicated that Mlt showed antibacterial activity in a longer time. Moreover, ZnO/Mlt had better antibacterial activity that could be attributed to the presence of ZnO. The results are similar to the ones reported by Ehsani et al.^24^, demonstrating that loading ZnO on clays could improve antibacterial properties. ZnO shows antibacterial activity via the electrostatic activity between the negative charge of bacterial cells and the positive charge of ZnO nanoparticles. The results show a positive interaction between Mlt and ZnO for antibacterial activity. ZnO/Mlt/Chsn-NC had better antibacterial activity than ZnO/Mlt that could be attributed to the antibacterial activity of chitosan. Chitosan is known to have antibacterial properties by mechanisms, including penetration in the cellular membrane^13,15,21^. In the current study, the release of LDH and the production of ROS were investigated. Based on findings, NCs penetrate into bacterial membrane integrity, disrupt it, and increase the release of LDH in bacteria. In addition, the produced ROS directly attacks bacterial membrane, destroys it, and finally causes cellular death. The results show that Mlt, ZnO, and Chsn have a synergism interaction effect on antibacterial activity. The effects of ZnO/Mlt/Chsn-NC were almost similar for both bacteria, revealing its efficiency for both bacteria.

### DPPH results

**Figure 8:**
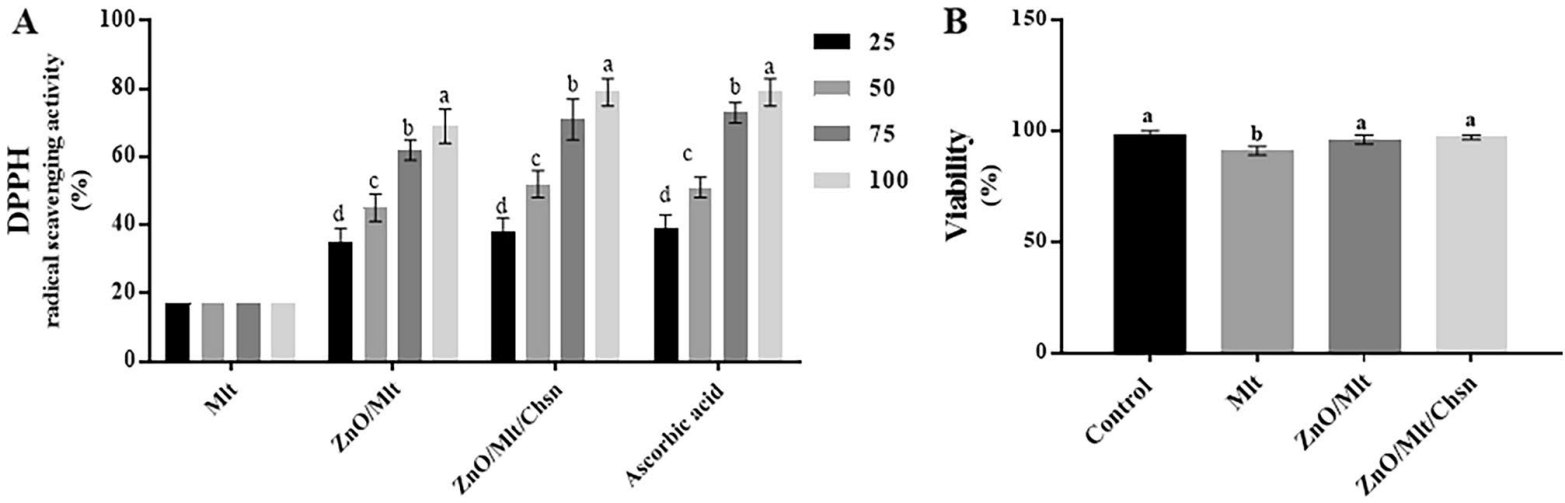
(**A**) 2,2-diphenyl-1-picrylhydrazyl radical scavenging results of Mlt, ZnO/Mlt-NC and ZnO/Mlt/Chsn-NC. (**B**) The results for the cytotoxicity of Mlt; ZnO/Mlt-NC and ZnO/Mlt/Chsn-NC.

Figure 8A presents the results of the antioxidant of the nanocomposites. The results showed that the lowest antioxidant activity was observed in the Mlt group; however, ZnO/Mlt showed better antioxidant activity. The highest antioxidant activity was observed in ZnO/Mlt/Chsn. Nanocomposites showed higher antioxidant activity with increased concentrations. The data analysis did not show any significant differences (P = 0.895) between ZnO/Mlt/Chsn and ascorbic acid for the antioxidant activity in the same concentrations. Anju et al.^12^ reported the antioxidant activity of Mlt via decreasing reactive oxygen species. Loading ZnO on Mlt improved antioxidant properties that could be attributed to the electron donation property of the oxygen atom in the ZnO nanomaterial^26^. Addition of chitosan to ZnO/Mlt increased antioxidant properties that might be attributed to the antioxidant properties of chitosan. Chitosan clears free radicals by mechanisms, such as adsorption, ion-exchange, and chelation^13,21^. In sum, antioxidant structures of ZnO, Mlt, and Chsn exhibit synergistic effects for antioxidant activity, since the values were higher for all concentrations in ZnO/Mlt/Chsn than for similar concentrations in Mlt and ZnO/Mlt.

### The results of cell viability

Figure 8B depicts the cell viability for different nanocomposites. The normalized viability of L929 cells showed that cells had better viability in the ZnO/Mlt/Chsn group than in the Mlt group. Similarly, Panandiker et al.^27^ showed that malachite at higher concentrations exhibited cytotoxicity owing to the involvement in antioxidant activity. The results showed that addition of ZnO decreased cytotoxicity that could be attributed to coating effects of ZnO. Gharehpapagh et al.^13^ showed that nanoparticles had lower cytotoxicity that coated the surface of the malachite and decreased its toxicity. Loading chitosan improved viability that might be attributed to coating effects of chitosan and its low toxicity. In sum, viability was almost 100.00% in ZnO/Mlt/Chsn, indicating it as a safe nanocomposite. Although Mlt showed cytotoxicity, it only lowered 10% viability.

### Wound area

**Figure 9:**
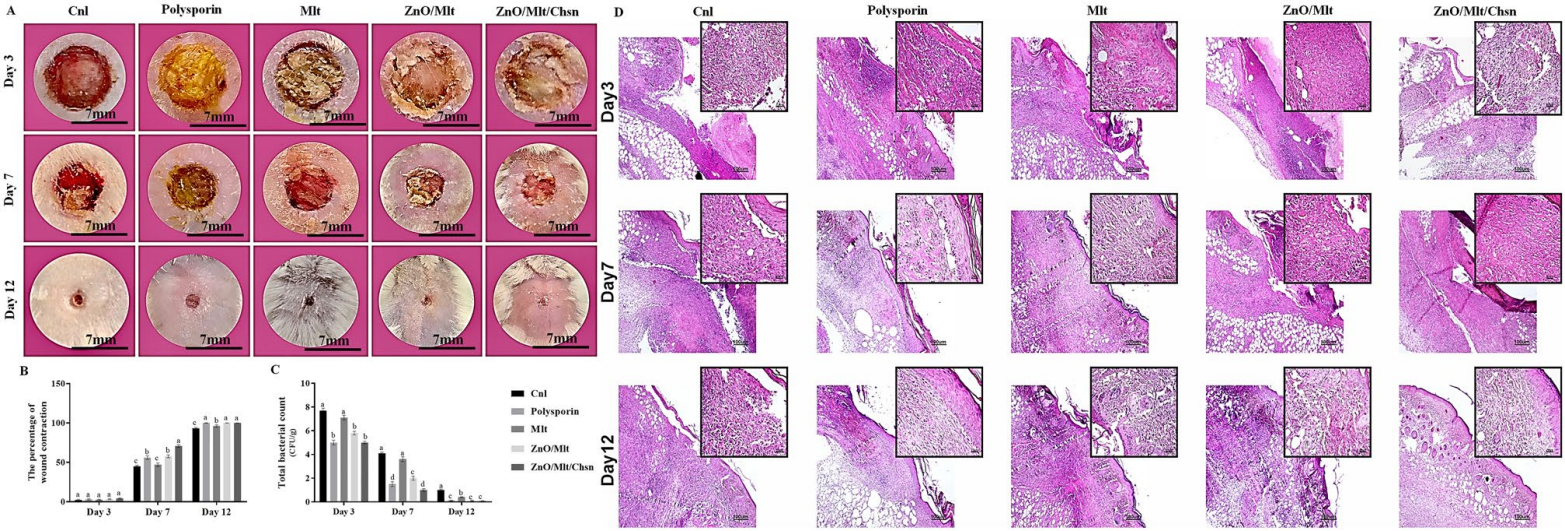
(**A**) Illustration of infected full-thickness wounds in the experimental group on different days after the wounding; (**B**) Wound area (mm^2^) for Cnl, polysporin, Mlt, ZnO/Mlt, and ZnO/Mlt/Chsn. The letters (a–d) indicate significant differences between the groups on the same day; (**C**) Total bacterial count (CFU/g) for Mlt, ZnO/Mlt, and ZnO/Mlt/Chsn. The letters (a–e) indicate significant differences between the groups on the same day. (**D**) Representative photomicrographs stained with the hemotoxiline and eosin staining for the wound area in Cnl, polysporin, Mlt, ZnO/Mlt, and ZnO/Mlt/Chsn groups.

Figure 9A,B shows the wound contraction (%) in different groups on days 3, 7, and 12. Data analysis did not show significant differences (*P* = 0.925) between the groups on day 3. The highest wound contraction was observed in the ZnO/Mlt/Chsn group than in the other groups on day 7 (*P* = 0.0001). The mice in polysporin and ZnO/Mlt exhibited similar wound contraction on day 7 (*P* = 0.485). The lowest contraction was observed in Mlt and Cnl groups on day 7. The mice in the Cnl group showed the lowest wound contraction on day 12. The mice exhibited a higher wound contraction in the Mlt group than in the Cnl group on day 12 (*P* = 0.025). The results indicate that Mlt has poor wound healing activity. Studies have not reported the wound healing activity of Mlt. However, ZnO/Mlt/Chsn revealed good wound healing activity and could compete with the standard ointment of polysporin. Studies have shown synergism interaction effects between ZnO and Chsn for the wound contraction^24^. ZnO and Chsn have antibacterial properties and improve histological parameters, such as edema, fibroblast, and collagen. The results suggest that ZnO/Mlt/Chsn is an appropriate for wound healing activity.

### Bacterial colony counts on wound surfaces

The results of the total bacterial count in Fig. 9C showed the highest total bacterial count in the Cnl group on all days. The mice in had a lower total bacterial count in the Mlt group than in the Cnl group on all days. Other groups had a lower total bacterial count. The results did not show significant differences between polysporin and ZnO/Mlt/Chsn ointment on all days. In other words, the similar efficiency for both agents reveals that ZnO/Mlt/Chsn ointment could compete with the commercial antibiotic of polysporin. The results of antibacterial activity are consistent with the results of the in vitro section. The mechanism antibacterial of the nanocomposites was previously explained. The results are in agreement with other studies indicating that ZnO-based structures modulate the microenvironments of bacteria to inhibit infection^28^.

### LPS results

**Figure 10:**
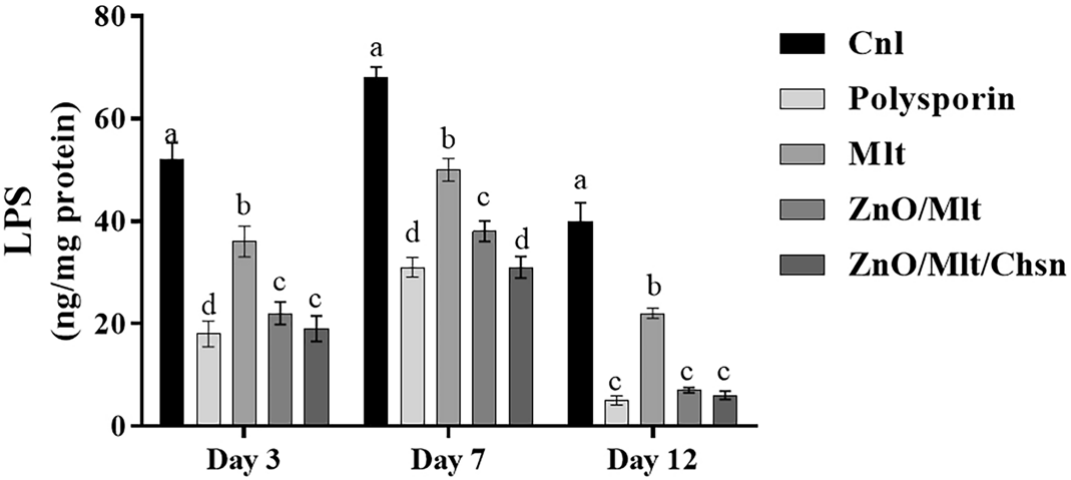
The results of the Lipoprotein polysaccharide concentration. The letters (a–e) indicate significant differences between the groups on the same day.

Figure 10 presents the results of the LPS concentration. The results showed the highest concentrations in the Cnl group on all days, and the lowest concentrations were observed in polysporin and ZnO/Mlt/Chsn groups. The mice treated with ZnO/Mlt had a lower concentration than that of the Cnl group on all days. LPS promotes the production of M1 macrophage and plays a crucial role in increasing the inflammation^15^. The findings show that prepared ointments decrease the inflammation, and the results are consistent with the results of the antibacterial section.

### Pathology results

**Figure 11:**
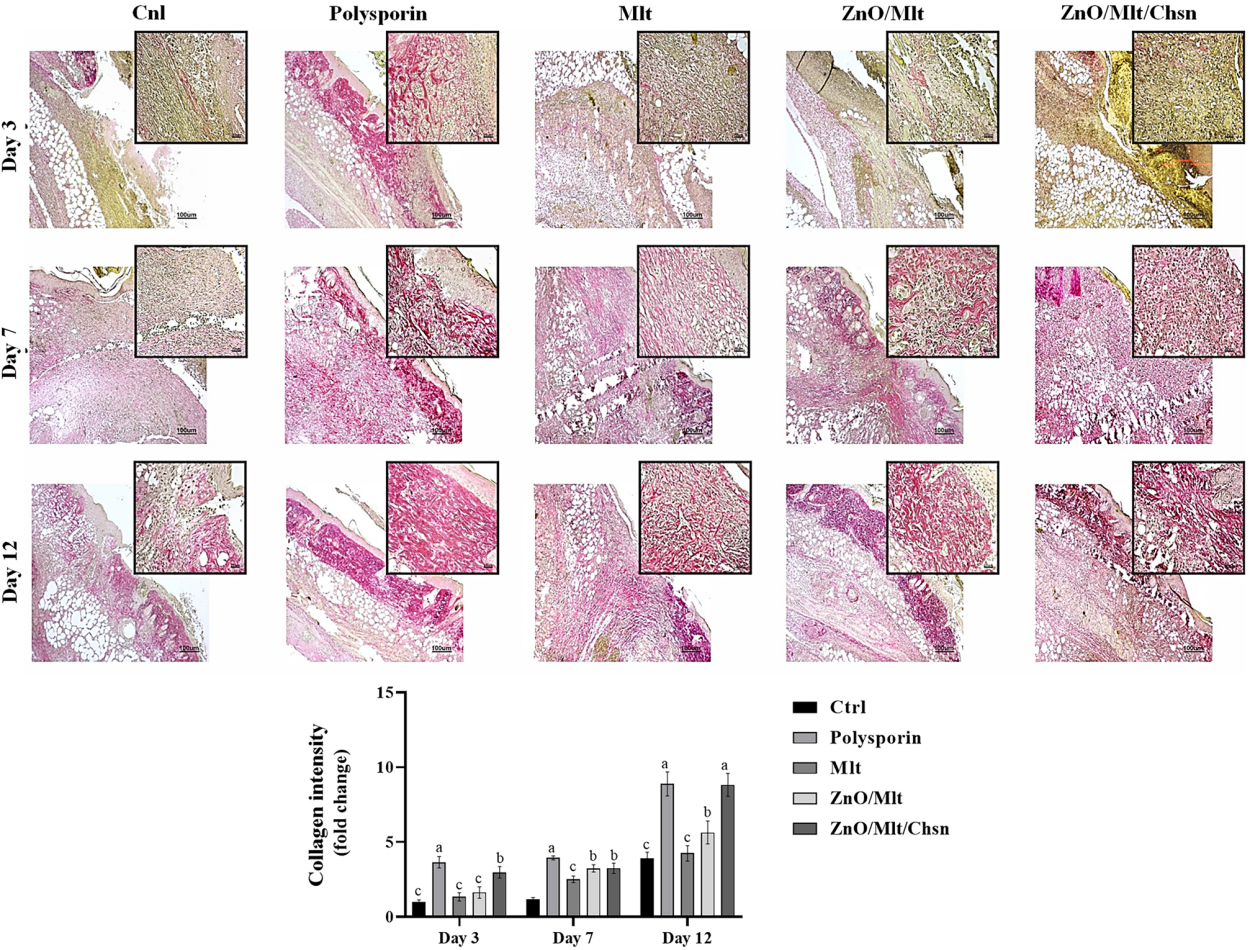
Representative photomicrographs on days 3, 7, and 14 stained with the Sirius red staining for the wound area in Cnl, polysporin, Mlt, ZnO/Mlt, and ZnO/Mlt/Chsn groups. See the collagen synthesis and well-recovered epidermis and dermis of the wound in ZnO/Mlt and ZnO/Mlt/Chsn versus the other groups.

Table 2 shows the effects of Mlt, ZnO/Mlt, and ZnO/Mlt/Chsn ointments on pathology parameters. The treated mice had lower edema and immune cells than the mice in the Cnl group on all days, except for the mice treated with Mlt ointment. The administration of ointments decreased immune cells and edema on all days. The number of vessels and fibroblast cells was significantly higher in the mice treated with polysporin, ZnO/Mlt, ZnO/Mlt/Chsn, and polysporin ointments than in the control mice on days 3 and 7 (Fig. 9D). Collagen scores were significantly (*P* < 0.05) higher in the mice treated with polysporin and ZnO/Mlt/Chn on day 12 (Fig. 11). All the mice had higher collagen than Ctrl mice on day 7 (*P* < 0.05). Epithelium did not have any thickness on day 3. The results indicated that the epithelium scores were significantly higher in the mice treated with polysporin and ZnO/Mlt, ZnO/Mlt/Chsn than in the control group on day 12.

**Table 2:** The effects of Mlt, ZnO/Mlt, and ZnO/Mlt/Chsn nanocomposite on histopathological parameters.

	Edema	Immune cells	Vessels	Fibroblast	Epithelium
Cnl	3.85 ± 0.32	38.80 ± 3.88	2.15 ± 0.66	12.00 ± 1.95	–
Polysporin	2.60 ± 0.25^##^	23.90 ± 1.77^###^	4.3 ± 1.54^##^	35.5 ± 1.15^##^	–
Mlt	3.70 ± 0.40	34.5 ± 1.05	2.9 ± 0.62	11.3 ± 1.1	–
ZnO/Mlt	2.65 ± 0.41^##^	29.00 ± 3.13^###^	3.80 ± 1.3^##^	30.7 ± 1.33^##^	–
ZnO/Mlt/Chsn	2.80 ± 0.25^##^	25.50 ± 3.19^###^	4.4 ± 1.1^##^	38.9 ± 1.00^##^	–
**Day 7**					
Cnl	2.85 ± 0.13	33.5 ± 3.31	8.7 ± 1.77	55.5 ± 4.32	–
Polysporin	0.00 ± 0.00^##^	11.95 ± 1.15^###^	14.00 ± 1.44^##^	90.5 ± 6.56^###^	0.01 ± 0.01
Mlt	1.67 ± 0.42^#^	17.25 ± 1.70^##^	9.9 ± 3.44	70.9 ± 3.99^##^	–
ZnO/Mlt	0.00 ± 0.00^##^	12.00 ± 1.45^###^	13.8 ± 1.14^##^	90.9 ± 3.55^###^	0.01 ± 0.02
ZnO/Mlt/Chsn	0.00 ± 0.00^##^	10.1 ± 1.00^###^	16.9 ± 1.14^###^	102.2 ± 3.91^###^	0.03 ± 0.03
**Day 12**					
Cnl	1.67 ± 0.42	7.90 ± 1.00	4.20 ± 0.33	67.4 ± 3.00	0.02 ± 0.05
Polysporin	0.00 ± 0.00^##^	1.50 ± 0.35^###^	3.30 ± 0.55^##^	35.2 ± 3.25^##^	0.04 ± 0.08^##^
Mlt	0.00 ± 0.00^##^	3.10 ± 0.54^##^	3.00 ± 0.32^##^	36.5 ± 3.78^##^	0.03 ± 0.02^#^
ZnO/Mlt	0.00 ± 0.00^##^	1.20 ± 0.45^###^	3.20 ± 0.10^##^	35.9 ± 3.20^##^	0.04 ± 0.07^##^
ZnO/Mlt/Chsn	0.00 ± 0.00^##^	1.30 ± 0.79^###^	3.60 ± 0.22^##^	44.00 ± 8.89^##^	0.05 ± 0.01^##^

The superscripts (#) show groups have significant differences (*P* < *0.05*) on same sampling day.

Malachite decreased edema and immune cells. In literature review, there was no paper investigating the effects of malachite on histological parameters. Notably, malachite shows weak antibacterial properties that decrease the edema and promote the proliferative phase. The results of histological parameters for ZnO are similar to the results reported by Ehsani et al.^24^. Anti-inflammatory properties and proliferative promoting effects are the mechanism suggested for the wound healing activity of ZnO nanoparticles^24^. Regarding the effects of chitosan as the wound healing structure, it must be stated that it causes to produce blood vessels, growth factors, and activating endothelial cells^15^. Chitosan and ZnO are antibacterial structures that accelerate wound healing, decrease the inflammation, and move the wound toward the proliferative phase. All the ointments increased collagen synthesis, thereby contracting the wound and closing it.

### The results of immunofluorescence staining and gene expression

**Figure 12:**
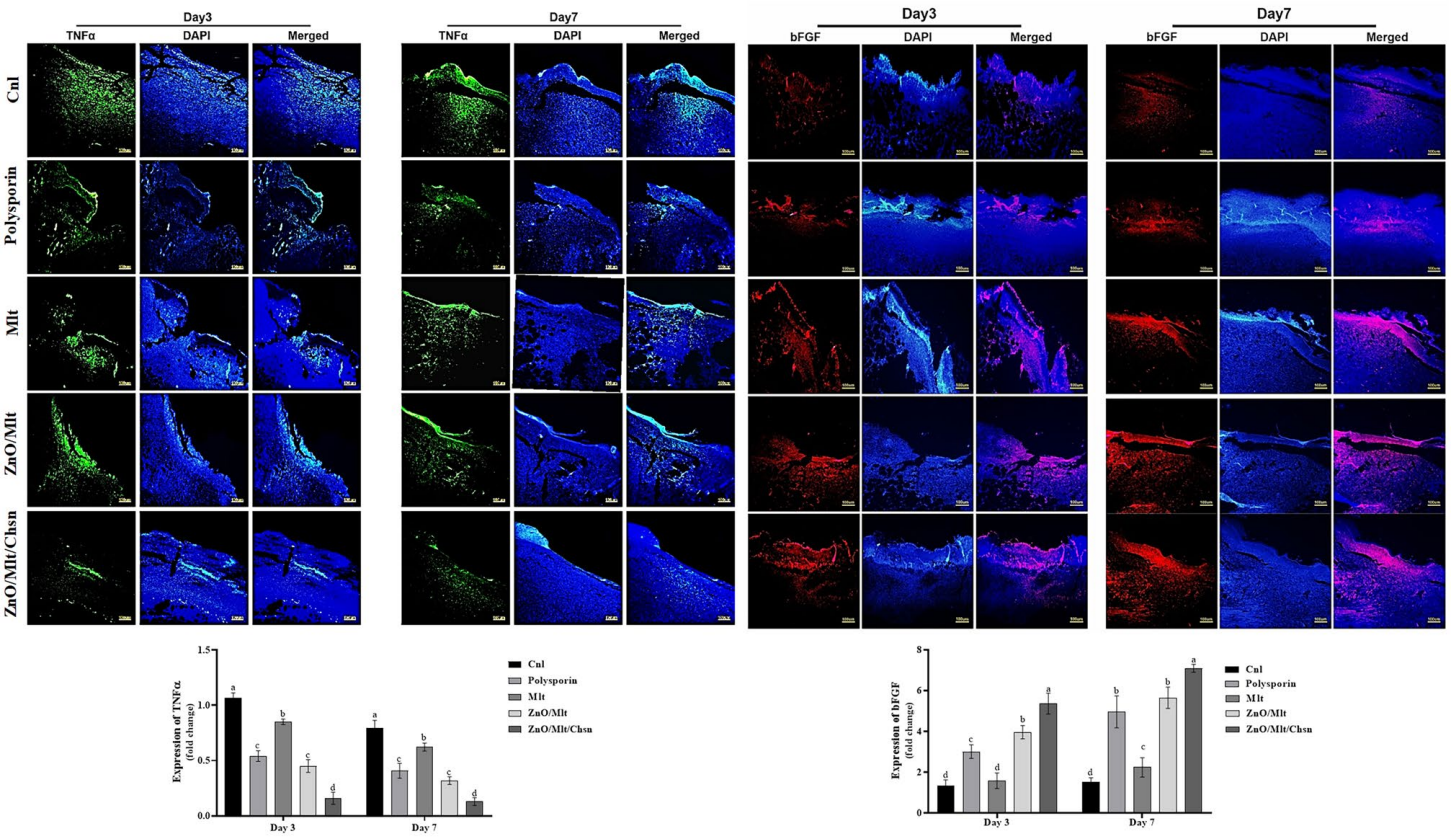
Cross-sectioning results of the protein expression of TNF-α and bFGF via the immunofluorescence staining on days 3 and 7 after wounding. See the TNF-α expression, which was significantly decreased, and the bFGF expression was significantly increased in the treated groups versus the control group. The different letters above each column indicate a difference in level (*P* < *0.05*) per sampling day.

**Figure 13:**
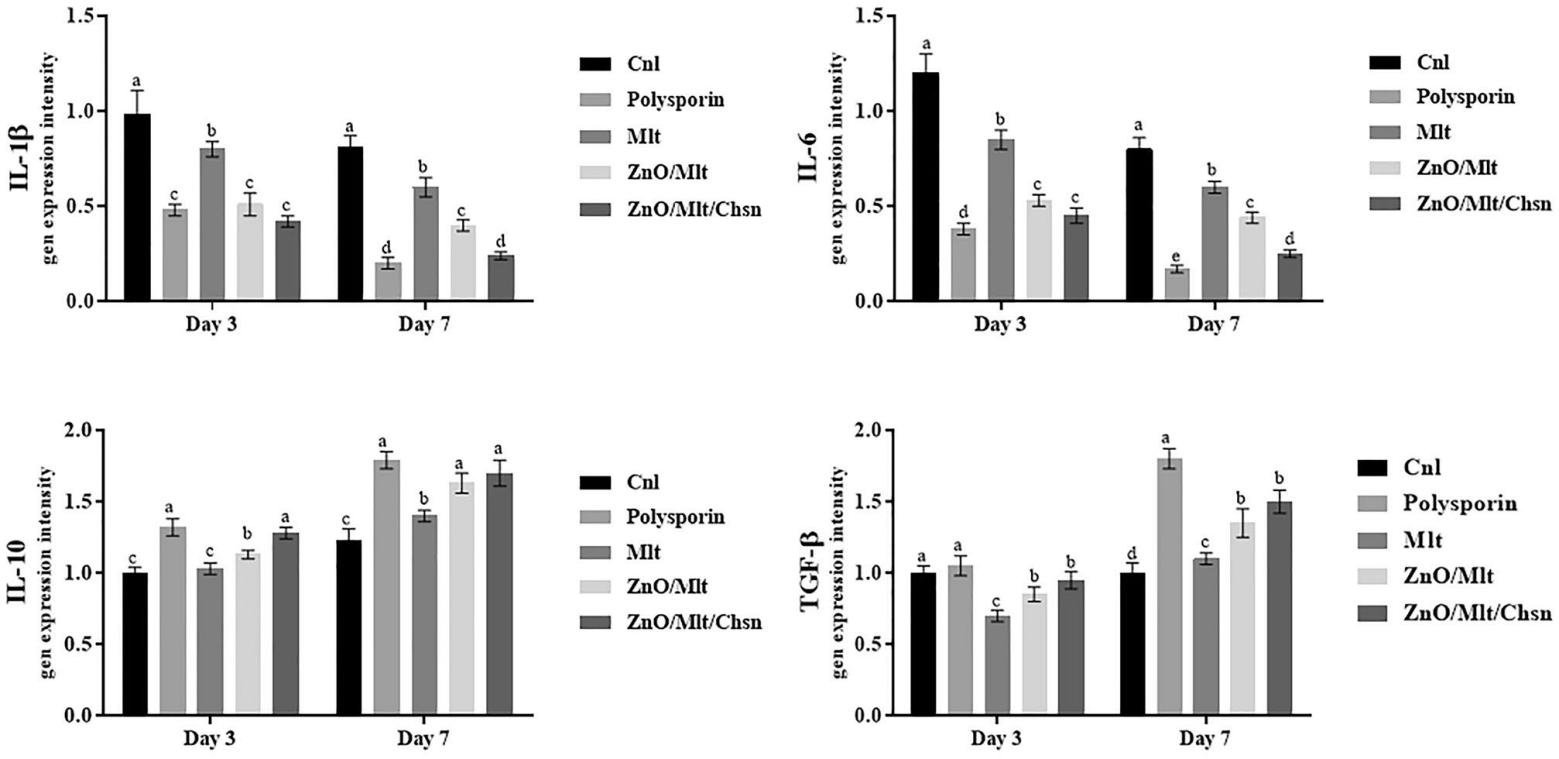
Relative IL-1β, IL-6, IL-10, and TGF-β mRNA expressions were measured by qRT-PCR using the 2_ΔΔCt method and b-actin as an internal control. The administration of ointments based on malachite reduced the expression of IL-1β and IL-6 compared to the control group. The administration of ZnO/Mlt and ZnO/Mlt/Chn ointments increased the expression of IL-10 on days 3 and 7 and TGF-β on day 7 compared to the control group. The lowest expression for IL-1β and the highest expression for IL-10 were observed in the mice treated with ZnO/Mlt/Chsn ointment and antibiotic. All data are presented in the mean ± SD. The different letters above each column indicate a difference in level (*P* < *0.05*) per sampling day.

The results of immunofluorescence staining showed that expression intensities of TNF-α and bFGF were significantly (*P* < 0.05) higher and lower in the control groups than in the other groups, respectively (Fig. 12). The administration of Mlt ointments significantly decreased the expression of TNF-α and increased the expression of bFGF. Compared to antibiotics, the mice treated with ZnO/Mlt and ZnO/Mlt/Chn ointments had higher and lower expression for bFGF and TNF-α, respectively. Indeed, Fig. 13 presents the results of the gene expressions of IL-1β, IL-6, IL-10, and TGF-β. The administration of ointments based on malachite reduced the expression of IL-1β and IL-6 (*P* < 0.05) compared to the control group. The administration of ZnO/Mlt and ZnO/Mlt/Chn ointments significantly (*P* < 0.05) increased the expression of IL-10 on days 3 and 7, and TGF-β on day 7 compared to the control group. The lowest expression for IL-1β and the highest expression for IL-10 were observed in the mice treated with ZnO/Mlt/Chsn ointment and polysporin.

The increased expression of IL-1β in the wound prolongs the pro-inflammatory condition and hinders the wound healing process^29^. IL-6 is a pro-inflammatory cytokine and causes the inflammation. TNF-α, as a pleiotropic cytokine produced by keratinocytes, macrophages, and mast cells, delays the wound healing process^31^. IL-10 acts in contrast to TNF-α and accelerates the wound healing process by reducing the inflammation^32^. Additionally, bFGF stimulates the proliferative phase and the participation in synthesis of new vessels^33^. TGF-β promotes the proliferative phase and accelerates the wound healing process^34,35^. Briefly, the increased expression of IL-1β, IL-6 and TNF-α increases the inflammation and retards the wound healing process. Based on the findings, ointments produced from malachite significantly decreased the expression of IL-1β, IL-6 and TNF-α and increased the expression of IL-10, bFGF and TGF-β. In other words, ointments precede the wound healing process by molecular mechanisms that could be attributed to their compounds, while commercial antibiotics show their effects by antibacterial activity.

## Conclusions

In sum, ZnO/Malachite nanocomposites were synthesized with the help of *M. pulegium* extract and its coated chitosan-derivatives to treat infected wounds. Physicochemical characterization of the prepared nanocomposites proved their successful synthesis. The prepared nanocomposite had antibacterial and antioxidant properties that accelerated the wound healing process and could compete with the standard ointment of polysporin. This study was conducted on infected wounds in mice, and the results cannot be used for other wounds in mice. Although, this study was conducted on mice, the potential results of the current study pave the way for clinical studies to investigate the effects of NCs on wounds and their uses in combination with other commercial ointments.

## Materials and methods

### Materials and methods

The chemical agents were of analytical grade. Zinc chloride, and medium molecular weight chitosan (190,000–310,000 Da., 167 75–85% deacetylated) were purchased from Merk and Sigma companies, respectively. Medicinal grade of malachite powder (free from hazardous elements) was prepared from Iranshid Co. Mashhad, Iran. To investigate morphological properties, EDX (MIRA3 FEG-SEM, Czech Republic) was used. Hydrodynamic diameter andsize distribution were evaluated, and zeta potential of the samples was investigated by DLS (Nanotrac Wave, Microtrac Co. USA). FTIR spectra (Shimadzu model FTIR 8101N spectrometer) was also used.

### Preparation of M. pulegium extract

Leaves of *M. pulegium* were collected from (in accordance with institutional and national guidelines) Botanical Garden of Tabriz University, washed with distilled water, shaken for 24 h, and placed in an ultrasound bath under reduced pressure. It was then exposed to microwave radiation and filtered to obtain the supernatant and stored in a refrigerator for further use based on other studies^13–15^.

### Biofabrication of ZnO/Mlt-NC, and ZnO/Mlt/Chsn-NC and preparation of ointments

To prepare ZnO/Mlt-NC, 0.20 g of ZnCl_2_ was mixed with 0.60 g of malachite powder in 75 ml of the extract, and then vigorously stirred at 65 °C for 12 h. The obtained raw product was then separated after centrifuging. This precipitate was washed with distilled water and ethyl alcohol to remove the remained ions and other impurities. Afterward, it was dried at 70 °C for 1 h to prepare ZnO/Mlt/Chsn-NC. One gram of ZnO/Mlt-NC powder was gradually added into 50 mL protonated chitosan solution (acetic acid 1% w/w) at room temperature, stirred for 24 h, and ZnO/Mlt/Chsn-NC was obtained. The therapeutic ointments (2% w/w) were formulated as reported by Mahmoudabadi et al.^14^. In sum, a certain amount of NCs (2 g) was mixed with 98 g base ointment to prepare NC ointments.

### In-vitro antibacterial assessments

#### Minimum inhibitory concentration (MIC) and minimum bactericidal concentration (MBC)

MIC and MBC methods were assessed as reported by Koeth et al.^36^, for *S. aureus* (ATCC 25923), and *P. aeruginosa* (ATCC 27853). In short, the wells were filled with 100 μL of fresh culture media (Mueller Hinton Agar; Merk Company- Germany). Then, 100 μL of the prepared nanomaterials were added to it at the highest concentration (64 µg/mL). Serial concentrations were prepared up to the tenth well. Additionally, 100 μL of the bacterial suspension (5 × 10^5^ CFU/mL) was added for all the wells except for the first well (negative control). The OD values were investigated in a wavelength of 630 nm after 24 h and at 37 °C. First well lack of turbidity was considered as MIC. To determine MBC, 10 µL was produced from wells lacking turbidity, negative wells, and positive wells with turbidity and then cultured in nutrient agar media. MBC was considered the minimum concentration of nanomaterial that inhibited bacteria growth after 24 h. The results were compared to the control antibiotics of bacitracin 10% (BidePharm Technology Company, Shanghai, China) for *S. aureus* and to polymixin B for *P. aeruginosa*.

#### Well method

The antimicrobial activity of the nanocomposites was evaluated with the agar diffusion method against *S. aureus* (ATCC 25923), and *P. aeruginosa* (ATCC 27853), as reported by Ehsani et al.^24^. Summary, 100 μL of 2% Mlt and ZnO/Mlt and ZnO/Mlt/Chsn were administrated into each well, and the diameter of the inhibition zone was measured at 37 °C after 24 h. The findings were compared to the control antibiotics of bacitracin for *S. aureus* and to polymixin B for *P. aeruginosa*.

#### Killing time assay

The growth curves of *S. aureus* and *P. aeruginosa* were investigated at different times as reported by Nguyen et al.^37^. Briefly, bacterial suspension solution was added to the Muller-Hinton media (Merk Company- Germany) culture containing nanocomposites with the same MIC concentrations and incubated in a shaker incubator at 37 °C, 200 rpm and for 24 h. Samples were collected at time intervals of 0, 1, 3, 6, 12 and 24 h and cultured on the Muller-Hinton media culture. The colony forming unit (CFU) of the organisms was investigated in triplicate, and a graph of the log CFU/mL was illustrated against time as reported by Nguyen et al.^37^.

#### Antibacterial mechanisms of NCs

To evaluate lactate dehydrogenase (LDH), bacterial cells (*S. aureus* and *P. aeruginosa*) were cultured on foils (Mlt and ZnO/Mlt and ZnO/Mlt/Chsn) located on inserted in 6-well plates (200 μL MH broth with 5 × 10^3^ cells per foil) and incubated for overnight. Cells without treatment were regarded as control. The samples were then transferred into microcentrifuge tubes and centrifuged at 1200 rpm for 5 min. Additionally, 100 µL of supernatants were transferred to 96-well plates, and 100 μL of the LDH assay mixture were included into each well. The plate was coated and incubated for 30 min at room temperature. The optical density of per well was registered at 450 nm on an ELISA reader, and the LDH leakage was reported as follows:

$$\left\{ {\left( {{\text{ODtest}}\, - \,{\text{ODblank}}} \right)\, - \,\left( {{\text{ODcontrol}}\, - \,{\text{ODblank}}} \right)/\left( {{\text{ODcontrol}}\, - \,{\text{ODblank}}} \right)} \right\} \times {1}00\% .$$

In this formula, OD test is the optical density of cells exposed to tested foils, and ODcontrol is the optical.

To evaluate reactive oxygen species (ROS), the cellular reactive oxygen species detection assay Kit (Abcam, Cambridge, UK) was used, and all the protocols were conducted based on the producer Company. All the conditions for culturing and incubation were similar to those for LDH. Following the incubation, the samples were transferred to microcentrifuge tubes and centrifuged at 1200 rpm for 5 min. Then, 100 µL of the supernatants were transferred into 96-well plates, and 100 μL of the diluted DCFDA were added to each well and incubated for an additional 45 min at 37 °C in the dark. Production of DCF was evaluated by fluorescence spectroscopy with an excitation wavelength at 485 nm and an emission wavelength at 535 nm on an ELISA reader.

### 2,2-diphenyl-1-picrylhydrazyl (DPPH) assay

2,2-diphenyl-1-picrylhydrazyl analysis was used to investigate antioxidant activity as reported by Gharehpapagh et al.^13^ using 0.1 mL Mlt, ZnO/Mlt, and ZnO/Mlt/Chsn diluted at concentrations of 25, 50, 75, and 100 μg/mL and 2.9 mL of 0.025 g/L DPPH in DMSO. In sum, after incubation at 25 °C for 45 min, the absorbance was then recorded at 517 nm using a PowerWave XS microplate spectrophotometer.

### Fibroblast cytotoxicity assay

To evaluate cytotoxicity, L929 cells (Pasteur Institute, Tehran, Iran) (1 × 10^5^–1 × 10^6^ cells/mL) were investigated as previously reported by Tominaga et al., (1999) using staining with calcein-AM and a fluorescence microscope (Optika, Italy) with 490 nm excitation to monitor viable cells for Mlt, ZnO/Mlt and ZnO/Mlt/Chsn^38^.

### In vivo infected wound activity evaluation

#### Experimental animals

The healthy BALB/c male mice (n = 90) aged 10–12 weeks with a weight of 28 ± 4 g were prepared. The animals had unlimited access to water and food. They were kept under lab conditions prior to the start of study as the conditioning period. The mice were kept under a lighting diet of 12 h. This study lasted for 12 days in accordance with the Iranian ethical guidelines for the use of animals. All the used protocols, such as study design, sample size, randomization, outcome measures, data analysis, experimental procedures, and report of results, were in agreement with the ARRIVE guidelines,. The protocols were approved by the Committee on the Ethics of Animal Experiments of Veterinary Faculty and the Islamic Azad University Council on Animal Care, Tehran, Iran (IR.IAU.SRB.REC.1399.180), and were in compliance with the Guide for the Care and Use of Laboratory Animals published by the US National Institutes of Health (NIH publication no.85-23, revised 1996). We declare that all methods were performed in accordance with the relevant guidelines and regulations.

#### The induction of infected wound

To induce wound, the general anesthesia was conducted by administrating the ketamine (50 mg/kg)/xylazine (10 mg/kg) cocktail intraperitoneally. After induction of anesthesia, the hair was shaved, and a circular full-thickness wound (with a diameter of 7 mm) was created by surgical cutaneous punch on the skin, and 0.5 McFarland Standard suspension in Muller-Hinton media (Merk Company- Germany) containing 10^7^ bacterial cell of *S. aureus*. Furthermore, *P. aeruginosa* was immediately administrated into the wound site as reported previously^15,24,39^. The treatment was started 24 h after inoculation of bacteria. The mice were divided into 5 groups and daily treated with 0.5 g of ointments of polysporin® (500 unit/g bacitracin and 10,000 unit/g polymixin B), Malachite (Mlt), ZnO/Mlt, and ZnO/Mlt/Chsn. A group was regarded as control (Cnl) and was not treated. To prepare ointments, 2 g Mlt, ZnO/Mlt, and ZnO/Mlt/Chsn were mixed with base ointment (soft white paraffin, Parsin Shimi Company-Iran), and 2%-ointments were prepared. The administration of ointments lasted for 12 days. The wound area was investigated with the help of a transparent paper over the wound as reported previously^14,15,24,39^. In summary, the wound site was measured by placing a transparent paper over the wound and tracing it using a graph sheet and formula calculations.

#### Bacterial colony counts on wound surfaces

Bacterial colony counts were investigated as reported previously^14,15,24,39^ with the help of sterile swab and cultured on plate count agar on days 3, 7 and 12, and the results were reported as CFU/g of granulation tissue. In short, 0.1 g of the sample was crushed, minced, and homogenized in a sterile mortar containing 10 mL of sterile saline and then diluted in tubes containing 9 mL of sterile saline. They were then cultured on plate count agar, incubated and the colonies were calculated.

#### Histopathological assessment

To investigate histopathological parameters, the samples were prepared from granulation tissue, along with 1–2 mm^2^ healthy tissue, fixed in formalin, embedded in paraffin, and stained with hematoxylin and eosin and sirius red staining, and they were investigated by a light microscope as reported previously on days 3, 7, and 12^14,15,24,39^. Two blinded pathologists investigated the samples by a microscope and reported the results.

#### Investigation of lipoprotein polysaccharide (LPS)

LPS was investigated as recommended by the producer company and using commercial kits (MyBioSourcere: Catalog No: MBS452438).

#### Immunofluorescence staining

Immunofluorescence staining was conducted as reported by previous study on days 3 and 7^40^. Briefly, skin samples were collected from the wound bed with 1–2 mm outside the wound. Paraffin-embedded tissue sections were dewaxed, rehydrated and immersed in citrate buffer (pH 7.4) for 20 min to retrieve antigen. The used antibodies included bFGF (sc-365106, Santa Cruz Biotechnology, Inc), TNF-α (Ab-6671, Abcam Company), Goat Anti-Mouse IgG (E-AB-1011, Elabscience Company-United States American), and Goat Anti-Rabbit IgG (E-AB-1014, Elabscience Company).

#### The gene expression by real-time PCR for the mRNA quantitation technique (qRT-PCR)

On days 3 and 7, the wound tissues were isolated as previously reported, and RNA was extracted by applying a standard TRIZOL procedure based on previous study^39^ and assessed by a spectrophotometer (260 nm and 260/280 = 1.8–2.0). The used primers included IL-1β, forward (5′-AACAAACCCTGCAGTGGTTCG-3′) and reverse (5′-AGCTGCTTCAGACACTTGCAC-3′); IL-6, forward (5′-CTTCCATCCAGTTGCCTTCTTG-3′) and reverse (5′- AATTAAGCCTCCGACTTGTGAAG-3′); IL-10, forward (5′-CCATCATGCCTGGCT CAGCAC-3′) and reverse (5′-TGTACTGGCCCCTGCTGATCC-3′); and TGF-β, forward (5-CCAAACGCCGAAGACTTATCC-3′) and reverse (5′-CTTATTACCGATGGGATGGGATAGCCC-3′). Finally, mRNA expressions were measured by quantitative real-time polymerase chain reaction using the 2_ΔΔCt method and b-actin as an internal control.

### Statistical analysis

The data were investigated for normality by the Shaipro-wilk test in the SPSS software (version 23). The data were normal for all the parameters excluding histopathological parameters. The data for histological parameters were analyzed by Kruskal–wallis, and other data were analyzed by the one-way analysis.

### Ethical approval

All the used protocols were approved by the Committee on the Ethics of Animal Experiments of Veterinary Faculty and the Islamic Azad University Council on Animal Care, Tehran, Iran (IR.IAU.SRB.REC.1399.180), and also by the US National Institutes of Health (NIH publication no. 85-23, revised 1996).

## Data Availability

The present study doesn’t have any data set. Corresponded and request for material should be addressed to Mohammad Reza Farahpour.

## Abbreviations

DPPH: 2,2-Diphenyl-1-picrylhydrazyl
Al_2_O_3_: Aluminium oxide
bFGF: Basic fibroblast growth factor
Chsn: Chitosan
CFU: Colony forming unit
Cnl: Control
Cu(CO_3_)_2_: Copper(II) carbonate
Cu(OH)_2_: Copper(II) hydroxide
DLS: Dynamic light scattering
EDX: Energy dispersive X-ray analysis
FESEM: Field emission scanning electron microscope
FT-IR: Fourier transform infrared spectroscopy
IL-1β: Interleukin-1β
IL-6: Interleukin-6
IL-10: Interleukin-10
Fe_2_O_3_: Iron(III) oxide or ferric oxide
LPS: Lipoprotein polysaccharide
Mlt: Malachite
*M. pulegium*: *Mentha pulegium*
MBC: Minimum bactericidal concentration
MIC: Minimum inhibitory concentration
NC: Nanocomposite
NPs: Nanoparticles
qRT-PCR: Real-time PCR for mRNA quantitation technique
SiO_2_: Silicon dioxide
*S. aureus*: *Staphylococcus aureus*
*P. aeruginosa*: *Pseudomonas aeruginosa*
XRD: X-ray diffraction
Map: Mapping
TGF-β: Transforming growth factor-β
TEM: Transmission electron microscopy
TNF-α: Tumor necrosis factor alpha
ZnO: Znic oxide
